# Organic and inorganic carbon and their stable isotopes in surface sediments of the Yellow River Estuary

**DOI:** 10.1038/s41598-018-29200-4

**Published:** 2018-07-17

**Authors:** Zhitong Yu, Xiujun Wang, Guangxuan Han, Xingqi Liu, Enlou Zhang

**Affiliations:** 1 College of Global Change and Earth System Science, Beijing Normal University, Beijing, 100875 China; 2 Yantai Institute of Coastal Zone Research, Chinese Academy of Sciences, Yantai, Shandong 264003 China; 3 College of Resource Environment and Tourism, Capital Normal University, Beijing, 100048 China; 4 State Key Laboratory of Lake Science and Environment, Nanjing Institute of Geography and Limnology, Chinese Academy of Sciences, Nanjing, 210008 China

## Abstract

Studying the carbon dynamics of estuarine sediment is crucial to understanding of carbon cycle in the coastal ocean. This study is to evaluate the mechanisms regulating the dynamics of organic (TOC) and inorganic carbon (TIC) in surface sediment of the Yellow River Estuary (YRE). Based on data of 15 surface sediment cores, we found that TIC (6.3–20.1 g kg^−1^) was much higher than TOC (0.2–4.4 g kg^−1^). Both TOC and TIC were generally higher to the north than to the south, primarily due to the differences in kinetic energy level (i.e., higher to the south). Our analysis suggested that TOC was mainly from marine sources in the YER, except in the southern shallow bay where approximately 75% of TOC was terrigenous. The overall low levels of TOC were due to profound resuspension that could cause enhanced decomposition. On the other hand, high levels of TIC resulted partly from higher rates of biological production, and partly from decomposition of TOC associated with sediment resuspension. The isotopic signiture in TIC seems to imply that the latter is dominant in forming more TIC in the YRE, and there may be transfer of OC to IC in the water column.

## Introduction

The rate of CO_2_ build-up in the atmosphere depends on the rate of fossil fuel combustion and the rate of CO_2_ uptake by the ocean and terrestrial biota. About half of the anthropogenic CO_2_ has been absorbed by land and ocean. Large rivers that connect the land and ocean may play an important role in the global carbon cycle^1,2^. On the one hand, river can transport a significant amount of dissolved and particulate carbon materials from the land to the ocean, which are subject to recycling and sedimentation in the estuaries, or further transportation to the marginal seas^3,4^. On the other hand, there may be high levels of nutrients in the river waters, which could enhance biological uptake of CO_2_ and subsequent carbon burial in the estuaries^5,6^.

The Yellow River, the second longest river in China following the Yangtze River, provides approximately 50% of the freshwater discharged into the Bohai Sea every year^7^. There were some studies on sedimentary organic carbon around the Yellow River Estuary (YRE), which were mainly conducted in the Yellow River Delta^1,8,9^ and in the shelf of the Bohai Sea^10–13^. Limited studies showed a large spatial variability (ranging from 0.7 to 7.7 g kg^−1^) in total organic carbon (TOC) in the YRE^14^, with the highest contribution (40–50%) of terrestrial organic carbon near the delta^11^. However, little is known about the TOC dynamics in the sediment for the transitional zone near the river mouth.

Limited studies of inorganic carbon dynamics have been conducted in the YRE. An earlier study showed that particulate inorganic carbon (1.8% ± 0.2%) was significantly higher than particulate organic carbon (0.5% ± 0.05%) in the water column of YRE^15^. A later analysis demonstrated that rate of CaCO_3_ precipitation was modestly higher than rate of biological production in the water columns of the estuary^16^. These findings suggest that there might be more inorganic carbon (TIC) than TOC accumulated in the sediment of the YRE. However, there is no evidence to support it because little is known on the magnitude and variability of TIC in the YRE. On the other hand, recent studies have showed that there was a large amount of carbonate in the soils of lower part of the Yellow River Basin, and higher level of carbonate was associated with high level of organic carbon^17,18^. One may expect a similar phenomenon in the sediment of the YRE.

As the world’s largest carrier of fluvial sediment, the Yellow River’s sediment load has continually decreased since the 1950s due to changes in water discharge and sediment concentration by anthropogenic changes^19^. On the other hand, climate change and human activities in the Yellow River basin have decreased fine sediment from the Loess Plateau and increased coarse sediment scouring from the lower river channel^20^. These changes may have profound impacts on the physical, biogeochemical and biological processes in the YRE. This study is the first to assess the dynamics of both TOC and TIC in the surface sediment of the YRE, focusing on the transitional zone near the river mouth^21^. The objective of this study is to test the hypothesis of more TIC than TOC accumulated in the sediment, and to explore the underlying mechanisms that regulate the variability of TOC and TIC in the YRE.

## Results

### Physical characteristics

**Figure 1:**
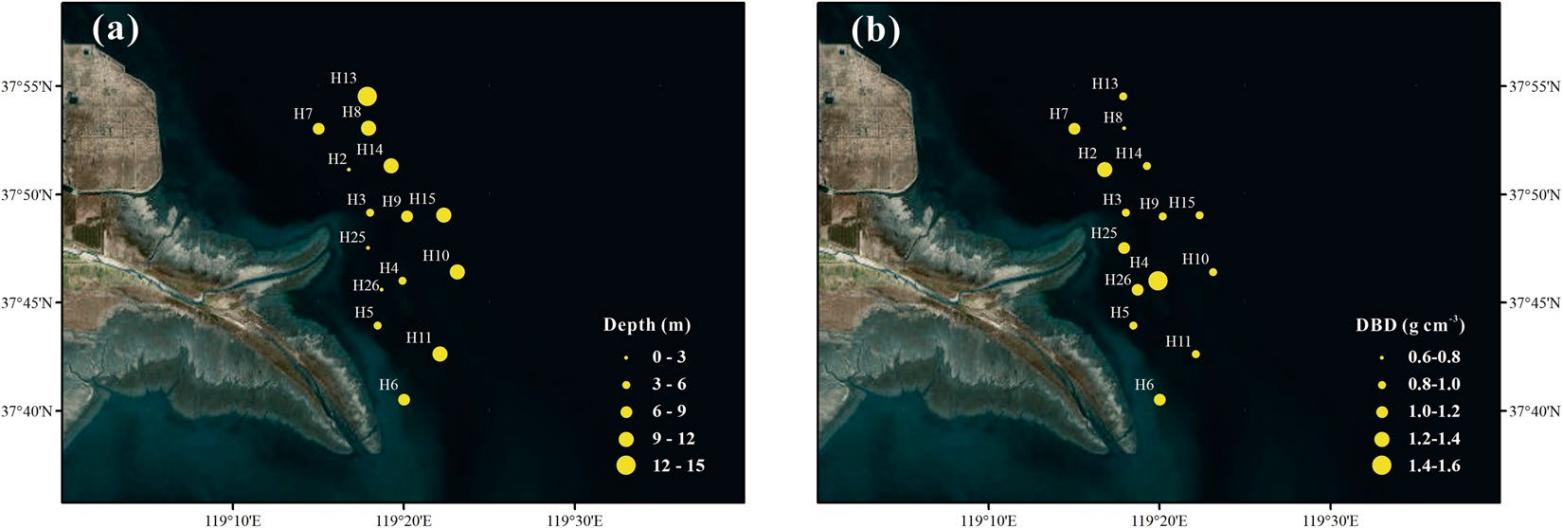
Spatial distributions of **(a)** depth (m) and **(b)** dry bulk density (DBD, g cm^−3^) in surface sediments of the Yellow River Estuary. The maps were generated by ArcGIS 10.2 (http://www.esri.com/arcgis/about-arcgis).

The sampling sites covered most parts of the YRE, with water depth ranging from 1.5 m to 13.5 m (Fig. 1a). Dry bulk density (DBD) ranged from 0.74 to 1.55 g cm^−3^, with an average of 1.02 g cm^−3^ (Table 1). Generally, DBD was much higher in the shallow water area than in the deep water region, presenting high values mainly occurred in the south and north sides near the river mouth (Fig. 1b).

**Table 1:** Means, standard deviation (SD) and coefficients of variation (CV) of the main variables.

		**DBD**	**d0.5**	**Clay**	**F-Silt**	**M-Silt**	**C-Silt**	**Sand**	**TN**	**TOC**	**TIC**	**C:N**	**δ** ^**13**^ **C**_**org**_	**δ** ^**13**^ **C**_**carb**_	**δ** ^**18**^ **O**_**carb**_
		**g cm** ^**−3**^	µm	%					**g kg** ^**−1**^				‰		
Sediment	Mean	1.02	34.2	6.1	34.8	13.7	20.9	24.5	0.36	2.3	14.1	6.3	−23.35	−4.36	−8.92
	SD	0.20	26.3	2.9	19.0	6.7	9.4	23.8	0.19	1.3	4.0	1.7	0.48	0.41	0.90
	CV	0.20	0.77	0.47	0.55	0.49	0.45	0.97	0.53	0.57	0.28	0.27	−0.02	−0.09	−0.10
Wetland Soil	Mean	/	28.2	4.7	31.1	25.8	25.8	12.5	0.77	8.9	12.9	10.8	−22.5	−4.0	−9.1
	SD	/	15.8	1.2	15.5	9.3	10.4	15.1	0.55	8.0	4.8	3.0	3.4	0.72	0.62
	CV	/	0.56	0.26	0.50	0.36	0.40	1.21	0.71	0.90	0.37	0.27	−0.15	−0.18	−0.07

Clay: <2 µm, F-Silt: 2–16 µm, M-Silt: 16–32 µm, C-Silt: 32–64 µm, Sand: >64 µm; C:N: TOC:TN.

**Figure 2:**
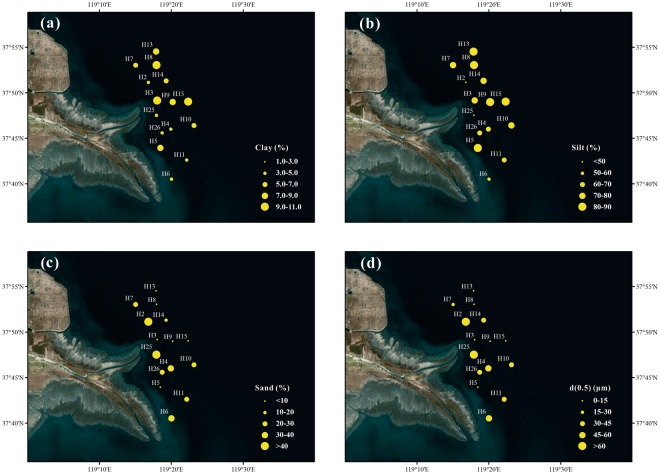
Distributions of **(a)** clay (%), **(b)** silt (%), **(c)** sand (%), **(d)** the median diameter (d(0.5), µm) in surface sediments of the Yellow River Estuary. The maps were generated by ArcGIS 10.2 (http://www.esri.com/arcgis/about-arcgis).

Figure 2 showed the spatial distributions of the main granulometric variables of the surface sediment. In general, clay content was low, ranging from 1.4 to 10.8% (Table 1), with relatively higher values in the northern part than in the southern part. The highest clay content was found near the north side of the river mouth, and the lowest at the mouth section. Silt content was much high (69.4 ± 21.1%), exhibiting similar spatial distribution with clay. On the other hand, the highest content of sand was found at the mouth (Fig. 2c), where clay and silt contents were lowest (Fig. 2a,b). As expected, the spatial distribution of d(0.5) was similar to that of sand, displaying the highest values in the shallow river mouth section and lowest in the southern bay, indicating strong hydrodynamic effect in the former and weak in the latter.

### Spatial distributions of TOC, TN, C:N and δ^13^C_org_

**Figure 3:**
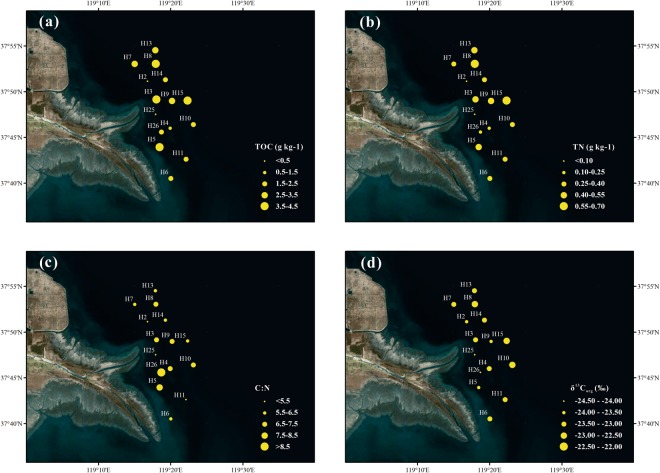
Spatial distributions of **(a)** TOC (g kg^−1^), **(b)** TN (g kg^−1^), **(c)** C:N, **(d)** δ^13^C_org_ (‰) in surface sediments of the Yellow River Estuary. The maps were generated by ArcGIS 10.2 (http://www.esri.com/arcgis/about-arcgis).

Concentration of TOC was highly variable, with higher values (3.2–4.4 g kg^−1^) in the northernmost section of the estuary and the east deep water area (Fig. 3a). There was also a high value of TOC in the bay, south of the river mouth. On the other hand, lower TOC concentration (0.2–1.4 g kg^−1^) was observed in the south section. Similarly, TN value varied largely, from 0.06 to 0.68 g kg^−1^, with the lowest at the shallow water area near the river mouth and the highest in the north deep water section (Fig. 3b). Overall, the spatial distribution of TN was similar to that of TOC, both showing higher values in the north and east deeper water area.

The C:N ratio ranged from 2.1 to 10.1 (Fig. 3c). In general, C:N ratio was higher in the shallow water part relative to the deep water part. The highest C:N ratio (8–10) was found in the southern bay, and the lowest in the shallow water area near the river mouth (<4.5). Figure 3d showed a considerable spatial variability in the δ^13^C_org_ values with a range from −24.26‰ to −22.66‰. The δ^13^C_org_ value was more negative near the river mouth and its adjacent south bay, and less negative far away from the river mouth and the coast line.

### Spatial distribution of TIC, δ^13^C_carb_ and δ^18^O_carb_

**Figure 4:**
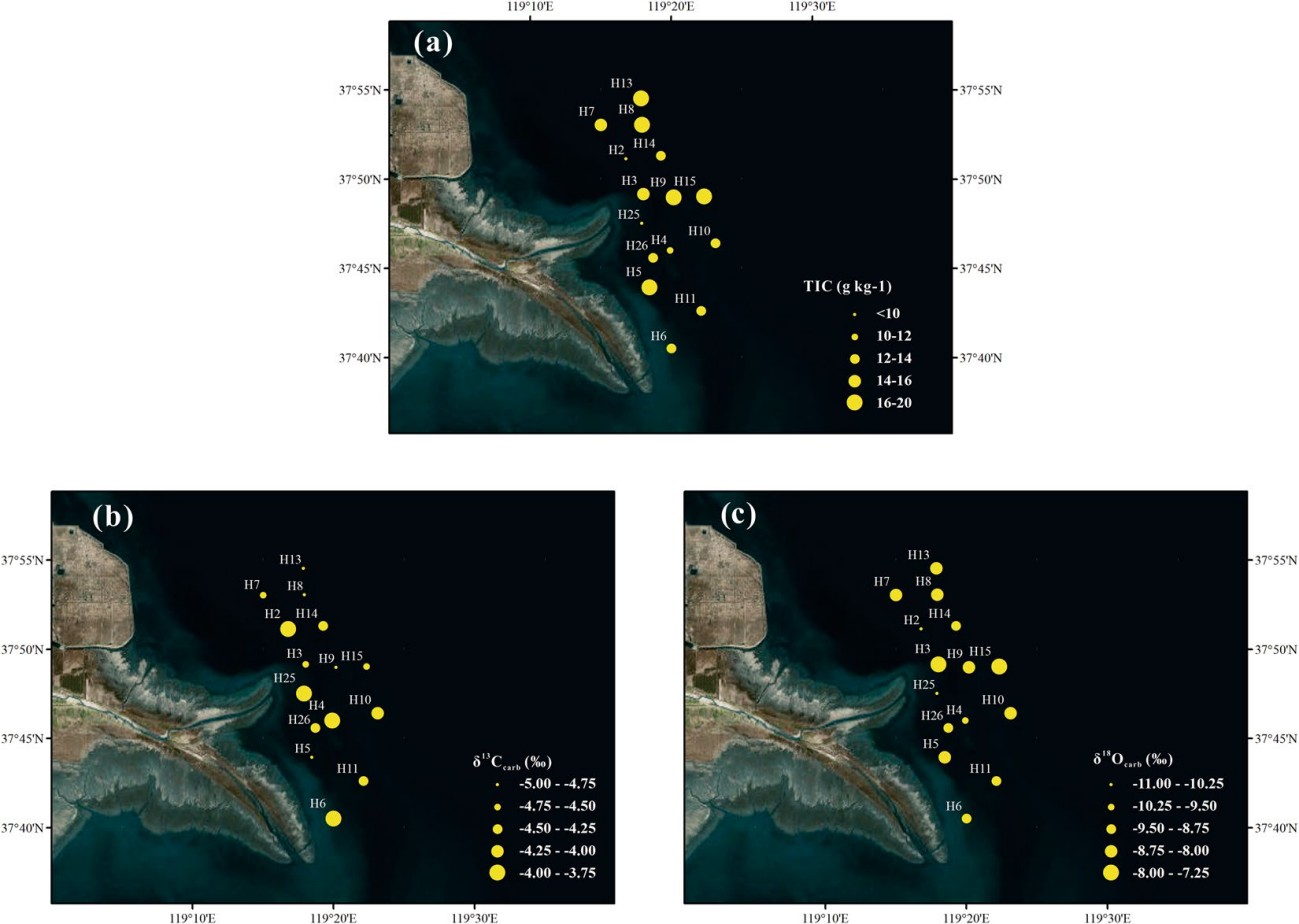
Spatial distributions of **(a)** TIC (g kg^−1^), **(b)** δ^13^C_carb_ (‰), and **(c)** δ^18^O_carb_ (‰) in surface sediments of the Yellow River Estuary. The maps were generated by ArcGIS 10.2 (http://www.esri.com/arcgis/about-arcgis).

There was a large spatial variation in TIC, as shown in Fig. 4a, ranging from 6.3 to 20.1 g kg^−1^, with higher concentration in the northern deep sea area (>17 g kg^−1^) away the mouth, and lower level in the south section (<13 g kg^−1^). Apparently, TIC also presented a high value in the north and east part. Overall, the spatial distribution of TIC was similar to that of TOC. The values of δ^13^C_carb_ and δ^18^O_carb_ ranged from −4.89‰ to −3.74‰ and −10.92‰ to −7.92‰, respectively (Table 1). Generally, the spatial distribution of δ^13^C_carb_ exhibited more negative values in the north and east deep sea area, which was opposite to that of δ^18^O_carb_ (Fig. 4b,c).

## Discussion

### Sources for TOC in the Yellow River Estuary

It is well known that human activities such as industrial and agricultural development would cause an increase in riverine input of nutrients and organic materials, leading to enhancements in estuary productivity and TOC burial in the sediment^22–24^. There was evidence that δ^13^C_org_ was less negative in the central Bohai Sea (−21‰ to −22‰) than in the nearshore (~−27‰)^11^, indicating more negative δ^13^C_org_ in terrigenous OC. Provided that the δ^13^C_org_ values ranged from −24.26‰ to −22.66‰, organic carbon in surface sediment of the YRE might be mainly from marine sources.

**Figure 5:**
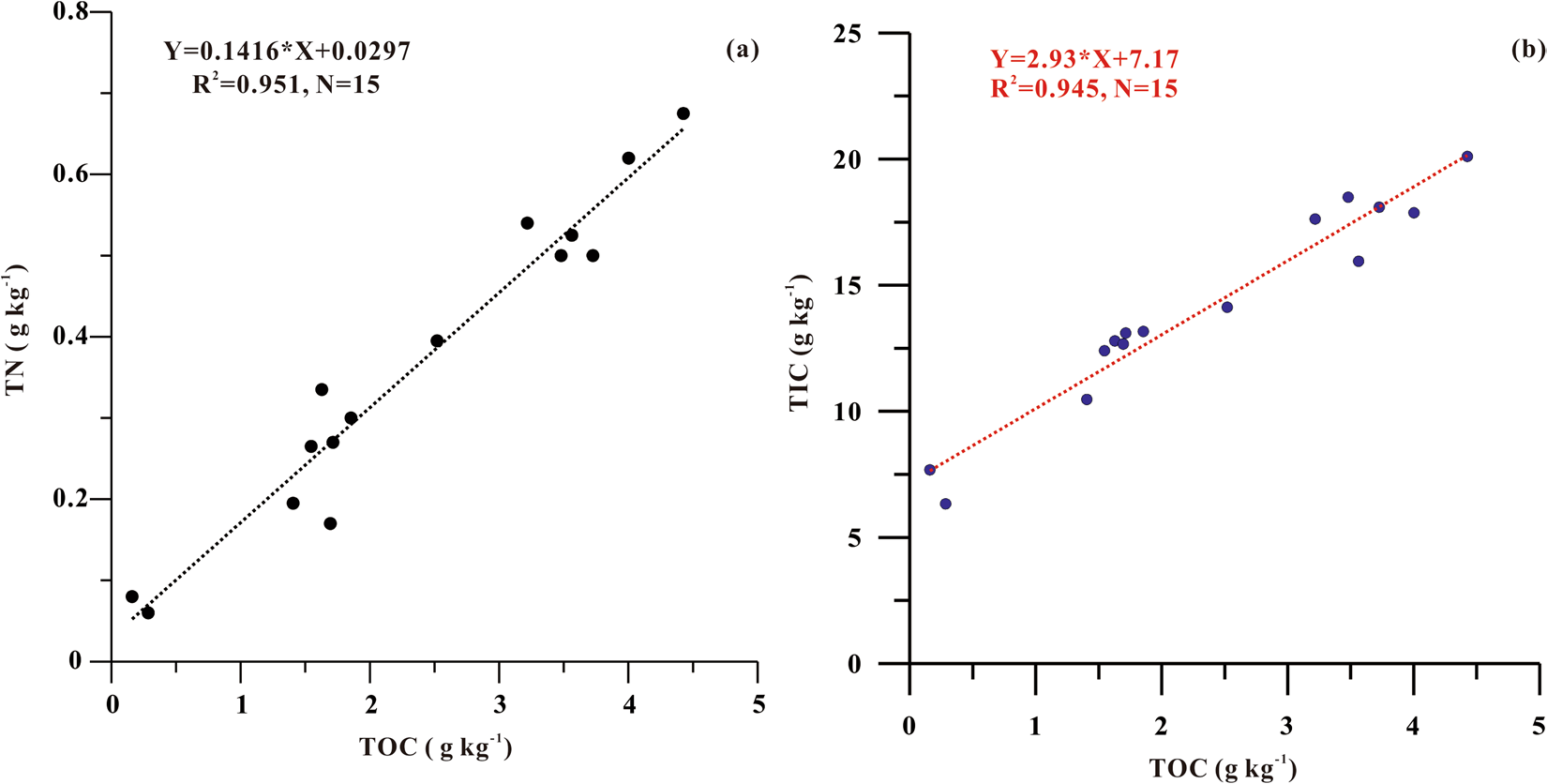
Relationship between **(a)** TOC and TN, **(b)** TOC and TIC in surface sediments of the Yellow River Estuary.

Since C:N ratio is significantly smaller in marine particles than in terrestrial organic matters, one may use a two-end-member mixing model to quantify different sources of OC; such approach has been widely applied in studies of wetland and lake sediments^25–27^, and offshore and marine sediments^28,29^. Given that TOC:TN ratio was lower than 5.5 g:g at some sites in the YRE, it was reasonable to assume that there were terrestrial inputs of inorganic nitrogen. There was a significant corelation between TN and TOC (Fig. 5a), with an intercept of 0.0297 g N kg^−1^. Following Schubert and Calvert^30^, we calculated total organic nitrogen (TON) concentration of each sample by subtracting 0.0297 g N kg^−1^ (the intercept) from TN. As shown in Table 2, TOC:TON ratio was low (<7.1) in most sections, illustrating that TOC was mainly autochthonous in the surface sediment the YRE. On the other hand, mean TOC:TON ratio was 9.5 in the southern shallow bay; such high C:N ratio together with relatively more negative δ^13^C_org_ value (Table 2) suggested that there might be a large amount of allochthonous OC sources in this section.

**Table 2:** Means of the variables in different sections.

Section	Depth (m)	DBD (g cm^−3^)	d(0.5) (μm)	TN (g kg^−1^)	TOC (g kg^−1^)	TIC (g kg^−1^)	TOC/TON	δ^13^C_org_ (‰)	δ^13^C_carb_ (‰)	δ^18^O_carb_ (‰)
North	10.8	0.88	20.2	0.47	3.0	16.2	6.8	−23.11	−4.70	−8.55
South	8.9	1.02	41.4	0.30	1.7	12.8	6.3	−23.04	−4.09	−8.96
Center	8.1	0.92	10.5	0.55	3.7	17.4	7.1	−23.15	−4.64	−8.03
Bay	3.5	1.21	33.4	0.29	2.3	13.7	9.5	−23.71	−4.31	−9.00

North: H7, H8, H13, H14; South: H6, H10, H11; Center: H3, H9,H15; Bay: H4, H5, H26.

To quantify the relative contributions of autochthonous and allochthonous OC in the surface sediments, we applied a two-end-member mixing model by using TOC:TON ratio, and assuming 6.6 mol:mol as the marine end-member. Using the average C:N ratio (10.8 g:g) from the soils collected near the river mouth (Table 1), we estimated that 75% of TOC was from soil OC source in the bay section, but only 12–28% in other sections of the YRE (Table 3). However, our approach could introduce bias or uncertainty due to the choice of end member value for soil C:N ratio. According to our recent study^31^, soil C:N ratio varied from 9.5 to 13.4 in the middle-lower parts of Yellow River Basin. If we chose 9.5 (or 13.4) as the soil C:N end member, the terrigenous contribution would be increased (or decreased) by 4–25%. Nevertheless, TOC in the surface sediment was primarily autochthonous in most parts of the YRE.

**Table 3:** Relative contributions (%) of marine and terrestrial sources using different soil C:N ratios as the end-member.

Section	Soil_C:N_ = 10.8^A^		Soil_C:N_ = 9.5^B^		Soil_C:N_ = 13.4^C^	
	Marine	Terrestrial	Marine	Terrestrial	Marine	Terrestrial
North	78	22	70	30	85	15
South	88	12	84	16	92	8
Center	72	28	62	38	81	19
Bay	25	75	0	100	50	50

A, B and C presented the soil C:N in our study, in the lower Yellow River Basin and in the Chinese Loess Plateau, respectively.

### TOC variability in the Yellow River Estuary

The magnitude and spatial distribution of TOC in estuarine sediment may reflect multiple and complex processes^10,32^. As shown in Fig. 2, the surface sediments were finer to the north than to the south. In general, coarser (finer) sediment particles usually indicated a stronger (weaker) water energy environment^33,34^. These analyses indicated that the relatively lower TOC values in the south section were attributable to higher kinetic energy level. On the other hand, a significantly positive relationship (r = 0.71, p < 0.01) between the δ^13^C_org_ value and water depth (Table 4) implied that the shallow sections in the YRE accumulated more terrigenous OC (with more negative δ^13^C_org_ values).

**Table 4:** Correlation coefficient (r) between various variables for the sediments.

	Depth	DBD	d0.5	Clay	Silt	Sand	TOC	TIC	δ^13^C_org_	δ^13^C_carb_
TOC	0.54^*^	−0.65^**^	−0.94^**^	0.97^**^	0.88^**^	−0.90^**^		0.97^**^	0.50	−0.90^**^
TIC	0.63^*^	−0.70^**^	−0.96^**^	0.93^**^	0.93^**^	−0.94^**^	0.97^**^		0.47	−0.93^**^
δ^13^C_org_	0.71^**^	−0.37	−0.55^*^	0.55^*^	0.53^*^	−0.54^*^	0.50	0.47		−0.32
δ^13^C_carb_	−0.54^*^	0.73^**^	0.90^**^	−0.88^**^	−0.85^**^	0.87^**^	−0.90^**^	−0.93^**^	−0.32	
δ^18^O_carb_	0.63^*^	−0.65^**^	−0.98^**^	0.94^**^	0.96^**^	−0.97^**^	0.91^**^	0.93^**^	0.63^*^	−0.84^**^

Significance of Pearson correlation is marked with one (p < 0.05) and two (p < 0.01) superscripts.

There is evidence that the magnitude and variability of OC is largely influenced by primary productivity, followed by sediment resuspension and riverine input in the Yellow-Bohai Sea^35^. In general, an increase of water productivity would cause enriched ^13^C in carbonate^36,37^. However, we found a significantly negative correlation (p < 0.01, Table 4) between TOC and δ^13^C_carb_ in the YRE, indicating that higher levels of TOC (with more negative δ^13^C_carb_) were not a result of local biological production. Given that sediment resuspension played a large role in regulating the spatial-temporal variability of POC in the Yellow-Bohai Sea^35,38^, we inferred that the current system would cause re-distribution of POC thus TOC in the surface sediment. Therefore, more OC could deposit in the north and east deep water area (with lower kinetic energy levels) in the YRE.

### Dynamics of TIC and underlying mechanisms

Concentration of TIC in the surface sediment of the YRE was relatively higher in the north section (16.2 g kg^−1^) than in the south section (12.8 g kg^−1^) (Table 2), which was consistent with TOC. As shown in Fig. 5b, there was a significantly positive correlation between TOC and TIC in the surface sediments in the YRE (r = 0.97, p < 0.01), implying a potential relationship between the two parameters. In general, OC production (i.e., uptake of CO_2_) can induce changes of chemical properties in the water column, which often leads to precipitation of carbonate^36,37,39^. Our analyses showed that the change ratio between TIC and TOC (i.e., the slope of 2.93 in Fig. 5b) in the surface sediment of the YRE was close to the ratio of 3.6 for IC:OC in particles in the water column by Gu, *et al*.^15^, indicating that the spatial variability of TIC might be driven by variability of POC.

While higher levels of TIC might be associated with higher levels of TOC, there was a big intercept (7.17 in Fig. 5b) for the TIC-TOC relationship in the surface sediment, suggesting that there were other processes of CaCO_3_ formation, which were not linked with biological production. If higher levels of TIC were a result of higher rates of biological production, one would expect an enrichment of ^13^C in carbonate; on the other hand, higher rate of respiration/decomposition would lead to depleted ^13^C in dissolved IC thus in carbonate^36,37^. The significantly negative relationship (p < 0.01) between δ^13^C_carb_ and TIC in the YRE (Table 4) indicated that higher levels of TIC (with more negative δ^13^C_carb_) might result from high rates of decomposition of OC. Given that both TIC and TOC had a significantly negative correlation (p < 0.01, Table 4) with δ^13^C_carb_ in the YRE, we speculated that there might be decomposition of TOC/POC associated with sediment resuspension, which would lead to an increase in dissolved IC thus promote carbonate precipitation and sedimentation.

### Comparisons with other studies

There have been many studies of TOC but only a few studies of TIC from the estuarine sediments. Overall, TOC levels are lower in the surface sediments in most estuaries in China, relative to those in the South and Southeast Asia^40,41^, Europe^42,43^, North America and South America^44,45^. In general, sedimentary TOC concentration is relatively lower in large river estuaries (e.g., the Yangtze River Estuary^46,47^ and Pearl River Estuary^48,49^) than in small river estuaries (e.g., the Luan River Estuary^50^, Licun Estuary^51^, Min River Estuary^52^ and GQ Estuary^53^), which indicating that weak hydrodynamic environment (in the small estuaries) was beneficial to accumulation of organic carbon^11,54^.

For the surface sediment near the river mouth in the YRE, TOC concentration was modestly lower in our study (0.2 to 4.4 g kg^−1^) than the previous reports of 0.7–7.7 g kg^−1^ ^14^ and <1 to 6.0 g kg^−1^ ^11^, which may be attributable to the decline in the Yellow River’s discharge over the past decade^19^. On the other hand, TOC levels near the Yellow River’s mouth were significantly lower than those in the other coastal areas of the Bohai Sea, e.g., north off the YRE (2.6–17.2 g kg^−1^)^55^ and the Laizhou Bay (5.7–12.8 g kg^−1^)^13^, which may reflect the different influences of kinetic energy level and terrigenous inputs.

The surface sediments contained much lower TOC in the YRE than other large estuaries in China (i.e., Yangtze River Estuary^46,47^ and Pearl River Estuary^48,49^). Interestingly, the primary productivity in the YRE was higher than that in the Yangtze River Estuary^56,57^. On the other hand, our recent study indicated that POC in the Yellow River Estuary was comparable to that in the Yangtze River Estuary, and there was profound, nearly year-around sediment resuspension in the Yellow-Bohai Sea particularly in the shallow sections^38^, implying that surface sediment was subject to frequent disturbing, transportation and recycling thus decomposition, which might be partly responsible for the lower TOC levels in the YRE.

However, the YRE had much higher TIC values than those (3.3–8.2 g kg^−1^) in the Cochin Estuary^40^, Vellar and Coleroon Estuary^58^, and Chilika Lagoon^41^ of the South Asia. The large difference may be attributable to factors such as water quality, net biological production and respiration, and sediment resuspension processes^59–61^. For example, the conditions with rich calcium and magnesium ions and strong water exchange between salty and fresh waters would lead to much more carbonate precipitation in the YRE^13,16^.

## Conclusions and Implications

To our best of knowledge, this study is the first to evaluate both TOC and TIC in the surface sediment of the YRE, and to explore the underlying processes determining the dynamics of TOC and TIC. We found that TIC concentration (6.3–20.1 g kg^−1^) was much higher than TOC (0.2–4.4 g kg^−1^), and both TOC and TIC were higher to the north (3.0 and 16.2 g kg^−1^) than to the south (1.7 and 12.8 g kg^−1^). The relatively lower TOC and TIC values in the south section were attributable to higher kinetic energy level. Our analyses indicate that TOC in surface sediment are mainly autochthonous except in the southern bay where approximately 75% of TOC is probably from terrigenous OC. Overall low levels of TOC in the surface sediment of YER are mainly due to the profound resuspension that can cause enhanced decomposition. On the other hand, higher levels of carbonate in surface sediment of the YRE result partly from higher rate of biological production, and partly from decomposition of POC/TOC associated with sediment resuspension. The isotopic signiture in TIC seems to imply that the latter is dominant in forming more TIC in the YRE, and there may be transfer of OC to IC in the water column. Further studies with integrative and quantitative approaches are needed not only to assess the spatial and temporal variations of major carbon forms in the water column and sediments, but also to quantify the contributions of various sources and transformations among the different carbon pools, which aims to better understand the carbon cycle in the YRE in the changing environment.

## Materials and Methods

### Site description

**Figure 6:**
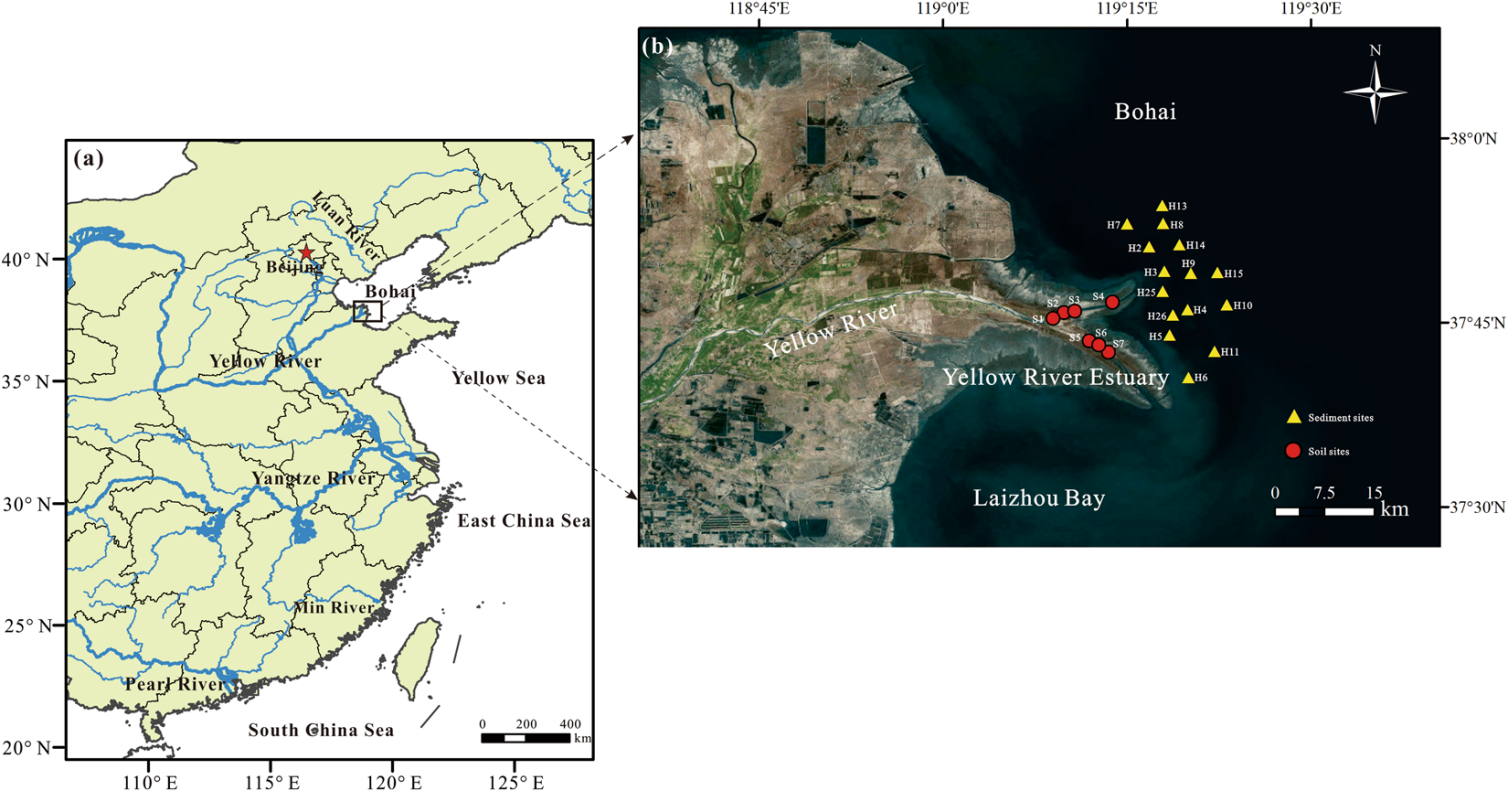
Map of **(a)** the large Chinese river-estuarine systems and **(b)** the Yellow River Estuary with the sampling sites. The figure was generated using ArcGIS 10.2 software (http://www.esri.com/arcgis/about-arcgis).

The YRE is a typical river-dominated estuary with weak tides, where has a warm-temperate continental monsoon climate with distinct seasons (Fig. 6). In the YRE, monthly water temperature is 4.1 °C in January and 26.7 °C in July, and annual wind speed ranges from 3.1 to 4.6 m s^−1^ in the estuary^62^. The estuary is characterized by a high sediment load (mainly composed of silt) in the water column, produced largely by the erosion from the China’s Loess Plateau. Most of the sediments discharged from the modern Yellow River mouth are trapped in the subaqueous delta or within 30 km of the delta front by gravity-driven underflow^9,63^. In recent decades, the annual water and sediment fluxes have declined dramatically, which is caused by regional climate change, reservoir construction, and irrigation-related withdrawals^16,19,62^.

### Field sampling and analyses

During October 2016, we collected 15 short sediment cores (H series) from the YRE using a Kajak gravity corer and 10 surface soil samples at 7 sites (S1–S7) along its upstream wetland (Fig. 6b). Each sediment core was carefully extruded and cut into 1-cm interval in the filed, and then placed in polyethylene bags which were kept on ice in a cooler during transport. In the laboratory, we took the top 2 cm sediment and surface soil samples, and then freeze-dried for 48 h before analyses.

Grain size was determined using a Malvern Mastersizer 2000 laser grain size analyzer. According to Yu *et al*. (2015), each sediment sample and soil sample (~0.5 g) was pretreated, in a water bath (at 60–80 °C), with 10–20 ml of 30% H_2_O_2_ to remove organic matter, and with 10–15 ml of 10% HCl to remove carbonates. The pretreated samples were then mixed with 2000 ml of deionized water, and centrifuged after 24 hours of standing. The solids were dispersed with 10 ml of 0.05 M (NaPO_3_)_6_, and then analyzed for grain size (between 0.02 and 2000 μm). The Malvern Mastersizer 2000 automatically outputs the median diameter d(0.5) (μm), the diameter at the 50th percentile of the distribution, and the percentages of clay (<2 μm), silt (2–64 μm) and sand (>64 μm) fractions.

Elemental analysis was measured using an Elemental Analyzer 3000 (Euro Vector, Italy) at the State Key Laboratory of Lake Science and Environment, Nanjing Institute of Geography and Limnology, Chinese Academy of Sciences. Freeze-dried samples were ground into a fine powder, then placed in tin capsules, weighed and packed carefully. For the analysis of TOC/soil OC, a ~0.3 g sample was pretreated with 5–10 ml 2 M HCl for 24 h at room temperature (to remove carbonate), and followed by washing with deionized water then drying overnight at 40–50 °C. Total carbon (TC) and total nitrogen (TN) were analyzed without pretreatment of HCl, and TIC/soil IC was calculated as the difference between TC and TOC/soil OC.

For the analyses of ^13^C in TOC/soil OC (δ^13^C_org_), approximately 0.2 g of the freeze-dried sample was pretreated with 5–10 ml 2 M HCl for 24 h at room temperature to remove carbonate, and then mixed with deionized water to bring the pH to 7, and dried at 40–50 °C before analyses. Each pre-treated sample was combusted in a Thermo elemental analyzer integrated with an isotope ratio mass spectrometer (Delta Plus XP, Thermo Finnigan MAT, Germany). Additionally, ^13^C and ^18^O in carbonate (δ^13^C_carb_ and δ^18^O_carb_) were measured following reaction with 100% phosphoric acid on a stable isotope ratio mass spectrometer (Thermo-Fisher MAT 253, Germany), at the Nanjing Institute of Geology and Paleontology, Chinese Academy of Sciences. All the isotope data were reported in the conventional delta notation relative to the Vienna Pee Dee Belemnite (VPDB). Analytical precision was 0.1‰ for δ^13^C_org_ and δ^13^C_carb_, and 0.2‰ for δ^18^O_carb_.

### Statistical methods and mapping

The p-value from the correlation analysis was derived from functions in SPSS statistics software (version 19, IBM, USA). A Pearson-test analysis was performed to determine the correlation’s significance. Spatial distribution maps were generated by ArcGIS 10.2 software (http://www.esri.com/arcgis/about-arcgis).
